# DART–Triple Quadrupole Mass Spectrometry Method for Multi-Target and Fast Detection of Adulterants in Saffron

**DOI:** 10.3390/metabo15060357

**Published:** 2025-05-28

**Authors:** Linda Monaci, Anna Luparelli, William Matteo Schirinzi, Laura Quintieri, Alexandre Verdu

**Affiliations:** 1 Institute of Biomembranes, Bioenergetics and Molecular Biotechnologies, National Research Council (IBIOM-CNR), Via G. Amendola, 122/O, 70126 Bari, Italy; 2 Institute of Sciences of Food Production, National Research Council (ISPA-CNR), Via G. Amendola, 122/O, 70126 Bari, Italy; annaluparelli@cnr.it (A.L.); williamschirinzi99@gmail.com (W.M.S.); laura.quintieri@cnr.it (L.Q.); 3 Bruker France S.A.S. Applied Mass Spectrometry, 34 Rue de l’Industrie, 67166 Wissembourg Cedex, France; alexandre.verdu@bruker.com

**Keywords:** food adulteration, food fraud, saffron, authenticity, mass spectrometry, DART-TQ-MS/MS, targeted method, chromatography-free

## Abstract

Saffron is a high-cost spice due to the specific conditions for optimal growth and because of being harvested by hand. The massive income from commercializing saffron substituted with other plant parts or low-cost spices makes this spice the main target of fraudsters. **Background**: Different methods have been developed for detecting saffron adulteration. Most of them are time consuming and complex, and in some types of analysis, the whole untargeted dataset is combined with advanced chemometric tools to differentiate authentic from non-authentic saffron. The official method, combining UV–vis spectroscopy and LC to determine the colour strength and the crocin content, is unable to detect saffron adulterants (safflower, marigold, or turmeric) added at a level lower than 20% (*w*/*w*). As a result, innovative approaches based on rapid, high-throughput methods for the identification of adulterated saffron samples are urgently demanded to counteract food frauds. **Methods**: This paper describes, for the first time, the development of a method combining Direct Analysis in Real Time (DART) with the triple quadrupole MS EVOQ based on the detection of specific MS/MS transitions, promoting a rapid, robust and chromatography-free method capable of monitoring safflower and turmeric adulteration in saffron. **Results**: The method proved to reach low LODs, allowing the determination of tiny amounts of turmeric and safflower powder in saffron as low as 3% and 5%, respectively, speeding up the whole analytical workflow and enabling us to perform 20 analyses in 10 min. Finally, the greenness of the method was also assessed according to the 0.88 score achieved by submitting it to the greenness calculator AGREE. **Conclusions**: Given its speed, simplicity, and robustness, this method stands out as a strong candidate for routine implementation in testing laboratories as a rapid screening tool to detect saffron adulteration with safflower or turmeric.

## 1. Introduction

Saffron (*Crocus sativus* L.) is one of the most valuable spices in the world. It derives from the dried stigmas of saffron flowers and is primarily used for cooking. Since ancient times, saffron has also been recognized in traditional medicine for its numerous health benefits, including antimicrobial, antispasmodic, aphrodisiac, antibacterial, antifungal, anti-inflammatory, and anticancer properties [1,2,3].

It is a source of bioactive constituents such as apocarotenoids, monoterpenoids, flavonoids, phenolic acids and phytosterols, widely investigated in experimental and clinical studies for a wide range of therapeutic effects [4].

Genuine saffron is meticulously hand-harvested according to regulated guidelines [5] and is highly valued for its intense colour and distinct aromatic profile, making it one of the most expensive spices globally. The high cost of saffron is largely due to the intensive manual labour involved in harvesting and trimming its stigmas. With each flower yielding only three stigmas, it does not account for the about 150,000 stigmas required to obtain 1 kg of spice [6].

The economic profit and high value of saffron are the reasons why saffron dealers in local markets try counterfeiting or adulterating saffron. Whole saffron is often adulterated in various ways to increase weight, improve appearance, or reduce production costs. This includes mixing it with older or condensed saffron, adding other parts of the saffron plant such as styles and stamens, or dipping it in substances like glycerin, olive oil, honey, or syrup. Chemical additives such as starch, various salts, and minerals are also used to alter its composition [7].

Additionally, adulteration can involve the incorporation of extraneous plant materials, the substitution with stigmas from Crocus species that lack colour and aroma, synthetic dyes, or lower-cost spices, all intended to replicate its sensory and visual characteristics, thereby compromising its authenticity and quality. In some cases, animal materials such as dried meat fibres are added [6,7].

The most common adulterants found in powdered saffron include turmeric powder, safflower powder, and paprika powder. In addition, saffron may be adulterated with artificial dyes such as Sudan I–IV and Rhodamine B, which are considered potentially genotoxic and carcinogenic. Powdered saffron is in general more at risk than the entire saffron and is vulnerable to adulteration with turmeric, paprika, salt, or synthetic dyes [6,7]. According to a recent report from the JRC about a campaign carried out in the period 2021–2022, it appeared that 11% of saffron in the spices category was the object of fraud, mainly due to adulteration by cheaper spices or artificial dyes [8]. Safflower was one of the low-cost spices most used for adulterating saffron.

Several chromatographic and spectroscopic methods are used for detecting adulterants in saffron. Although usually accurate and reliable, the main drawback of chromatographic methods is that they require a long analysis time and a high volume of organic solvents. For this reason, in the last few years efforts have been directed towards developing quicker and cheaper assays mainly based on UV-VIS spectroscopy for spice authentication [9,10]. Although up to 1% (*w*/*w*) of foreign material is permitted in third-class products, according to the ISO 3632 standards it was not possible to distinguish saffron adulterated with a low number of adulterants. For example, adulteration with safflower spice, marigold or turmeric up to 20% (*w*/*w*) was not detectable with ISO 3632 procedures [11].

Green techniques have been exploited to deliver a solvent-free and rapid method for food authenticity assessment; these techniques include infrared spectroscopy (IR), Fourier transform infrared spectroscopy (FT-IR) and laser-induced breakdown spectroscopy (LIBS). IR spectroscopy provides a versatile and cost-effective option for the high-throughput analysis of a diverse range of foods and herbs [12]. The applicability of IR spectroscopy for screening saffron adulteration with plant adulterants was also investigated by Chen et al. [13] using IR spectroscopic imaging, 2D correlation IR spectroscopy, and principal component analysis capable of distinguishing clusters containing crocetin and crocin (saffron) from other clusters without crocetin or crocin (the adulterant). Karimi et al. reported the capability of FTIR spectroscopy combined with appropriate chemometric techniques to detect and quantify six different artificial colorants in Iranian saffron [14].

Among the advanced spectroscopy-based methods, LIBS provided a rapid elemental analysis of the sample, can have application in food analysis [15,16], and was considered a promising tool for quantifying the level of adulteration in saffron samples with a simple and accurate process [17].

Nuclear magnetic resonance (NMR)-based metabolite fingerprinting may identify small differences existing between authentic and fraudulent products. This metabolomic approach has been explored to discriminate authentic saffron from commercial samples [18].

Among the molecular methods, considered high-throughput approaches, several Polymerase Chain reaction (PCR)-based methods have been used to detect saffron adulteration [19,20], including DNA sequence analysis [21,22] and Random Amplification of Polymorphic DNA-sequence-characterized amplified region (RAPD-SCAR) markers [23,24]. Although very sensitive, due to the intense sample prep required and the paucity of DNA present in this matrix, these methods are not very efficient in detecting traces of other plant parts as adulterants in saffron [25].

Recently, ambient pressure techniques have been exploited for food authenticity assessment. Applications to food authenticity study typically employ Direct Analysis in Real Time (DART) coupled to High Resolution MS (HRMS) for spices and saffron authentication [26]. While most of the studies so far published report the development of methods combining DART with HRMS for food authenticity assessment, only a few studies have explored the coupling between DART and low-resolution MS. A large number of applications of DART-MS to food authenticity have been published so far [26,27,28,29,30,31]. Untargeted metabolic fingerprinting using UPLC-HRMS has been exploited for saffron authentication/traceability according to the geographical origin along with the identification of some peculiar markers [32]. Elsewhere, a Liquid Chromatography-Q TOF-Mass Spectrometry (LC-MS) methodology has been described by Guijarro-Díez et al. [33] to assess the authenticity of saffron through the analysis of a group of glycosylated kaempferol derivatives proposed as novel authenticity markers.

In other research papers, the development of an integrated MS-based approach using DART technology combined with HRMS, both coupled either with an Orbitrap mass analyser or TOF-SIMS platform for assessing authentic saffron has been reported; both approaches are considered rapid and promising methods and propaedeutic to the identification of sensitive and reliable markers to safeguard saffron authenticity [34]. In general, the main advantage of metabolomics in food authentication lies in accurate mass measurements and its untargeted nature, which enables comprehensive comparative analyses and sample classification through chemometric tools. This approach allows for the characterisation of unknown samples via typical MS fingerprinting, the identification of emerging frauds, and the discovery of potential molecular markers for authenticity assessment. Despite the advantages offered by DART-HRMS-based methods, they are generally unsuitable for routine testing laboratories due to their high cost, complex operation and maintenance requirements. In stark contrast to that, triple quadrupole mass spectrometry (TQ-MS) offers a more practical solution, with affordable costs and the option to run multi-contaminant analysis by targeted methods, hence combining robustness, ease of use, and cost-effectiveness. Its superior sensitivity and selectivity in targeted quantification make it ideal for high-throughput screening in routine quality control settings.

This study presents, for the first time, the development of a fast, solvent-free, and robust quantitative MS/MS method, utilizing DART coupled with a triple quadrupole mass spectrometer (EVOQ DART-TQ+) for the determination of safflower and turmeric in saffron samples. Additionally, this work describes the first application of a targeted multi-reaction monitoring (MRM) approach using DART-TQ-MS for saffron authentication, representing a notable advancement by combining the rapid ambient-ionization capabilities of DART with the high sensitivity and selectivity of triple quadrupole mass spectrometry. The MRM approach offers key advantages in targeted mass spectrometric analysis, including high sensitivity, specificity, and reproducibility. Its analytical precision and low matrix interference, thanks to the double stage ions selection, make it ideal for routine applications. When combined with ambient-ionization techniques like DART, MRM further enhances analytical speed and throughput without compromising method performance or data quality.

The optimized analytical workflow in this paper is straightforward, requiring neither extensive sample preparation nor the use of toxic organic solvents. Overall, the method enables high-throughput analysis with a remarkably reduced run time—down to just 30 s per sample—making it a green, rapid, and efficient solution for routine screening.

## 2. Materials and Methods

### 2.1. Sample Preparation

50 mg of saffron, safflower and turmeric powder were individually extracted with 5 mL of EtOH/H_2_O (70/30; *v*/*v*) each. This composition was selected to achieve comprehensive spectral profiles enabling us to identify markers capable of distinguishing pure from adulterated samples based on the paper by Rubert et al. []. Moreover, a higher ethanol concentration was preferred to accelerate the evaporation step required for DART analysis. The mixture was shaken for 15 min at room temperature, centrifuged for 5 min at 13,000 rpm, and after filtration through PTFE filters (0.45 µm), it was diluted 1/1 with EtOH/H_2_O (70/30; *v*/*v*). A final volume of 2 µL was deposited on the DART 96 multi-wells card and left to dry for 5 min before its exposure to helium for DART–MS analysis. As a first step, to optimize the whole workflow and DART-MS parameters, a calibration curve, as described in Section 2.2, was prepared by diluting down the safflower extract with saffron extract to obtain several percentages of adulteration. This was preliminary to the preparation of the calibration curves obtained by mixing the respective powders (adulterant powder added to saffron powder) following detailed. For the final test, one authentic saffron sample and four blinded saffron samples (known to be adulterated) were extracted and analysed by the current multi-target method, and the final peak areas obtained were interpolated with the matrix-matched calibration curve equation to calculate the level of adulteration.

### 2.2. Preparation of Calibration Curves in Saffron Extracts Adulterated with Safflower Extracts

The safflower powder was extracted with EtOH/H_2_O mixture (70/30; *v*/*v*) and shaken for 15 min at room temperature. Likewise, saffron powder was extracted according to the same procedure and both extracts were mixed in order to cover the following levels: 10–20305070%(*w*/*w*) of safflower extract in saffron extract. Final mixtures at different fortification levels were analysed by DART-MS/MS in 5 replicates per each level to obtain the calibration curve.

### 2.3. Preparation of Calibration Curves in Saffron Powder Adulterated with Safflower and Turmeric Powder

For the preparation of the individual calibration curves, a fixed amount of safflower powder was added to the saffron powder in order to prepare a stock sample fortified at 500,000 ppm level with safflower powder (50%; *w*/*w*). Intermediate levels were produced by serial dilutions of the stock solution fortified at 50% with the saffron extract to cover the concentration levels 2.551020 and 50% (*w*/*w*). Pure saffron samples adulterated with turmeric at different percentages (2.55102050%; *w*/*w*) were prepared by using the same procedure as described for safflower.

### 2.4. Construction of Calibration Curves with Internal Standard

Caffeine, a molecule not expected to be present in saffron and its adulterating low-cost spices, was employed as an internal standard due to its stability and ionization features that makes it an ideal molecule for mass spectrometric detection. With the aim to obtain clean ion traces by reducing matrix interferences and to decrease variability of the analysis that might appear due to instrumental fluctuation, specific ion transitions were optimized and selected to build up the MRM method that included exclusive transitions for caffeine (as IS) in addition to selected transitions optimized for the adulterating compounds, safflower and turmeric. To correct signal deviation due to instrumental fluctuations, a same volume of caffeine standard solution at 0.2 µg/mL concentration was added to each saffron extract containing increasing concentrations of adulterant (either safflower or turmeric) before running DART-MS analysis; the obtained peak areas were finally corrected for the caffeine signal. The caffeine transition 196/138 was monitored and added to the multi-target MS/MS analysis.

### 2.5. DART-MS Analysis

The EVOQ DART-TQ^+^ mass spectrometer was purchased from Bruker (Bruker Daltonics GmbH & Co. KG, Fahrenheitstraße 4, 28359 Bremen, Germany) and was used for saffron analysis.

**Figure 1:**
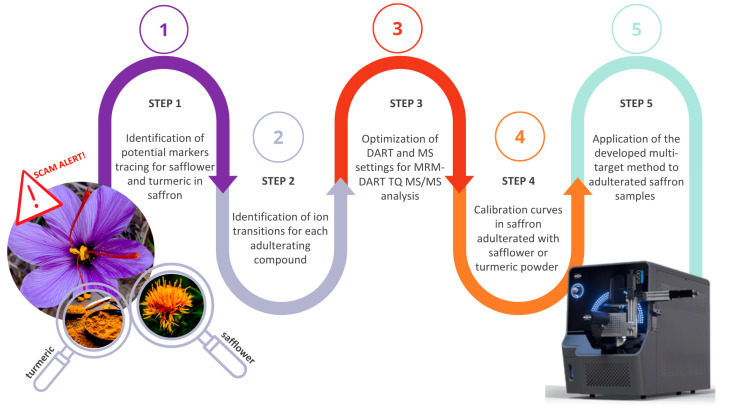
Key steps for the optimization of the DART-MS/MS method for the detection of safflower and turmeric in saffron.

The experimental workflow followed for method development is shown in Figure 1 and is briefly described below.

*DART sample introduction*: Three different modes were tested: Linear/Circular Scanning/Jumpshot. Circular scanning mode was selected as the preferred mode in terms of highest reproducibility observed among repetitive injections. Other parameters like motor speed 0.25 mm/s and scan diameter 2 mm were used as suggested by the manufacturer. Ion source parameters: grid cone voltage: 40 V; cone temperature: 350 °C; cone gas pressure: 20 PSI; DART temperature: 300 °C. Gas time program 6 s.

Cone and DART temperature were experimentally optimized to achieve optimal ionization efficiency and sensitivity for the target analytes by testing the values of 250 °C, 300 °C, 350 °C and 200 °C, 250 °C, 300 °C, respectively. The temperature was selected based on the best compromise found between maximizing ionization while minimizing thermal degradation of saffron components. The gas flow rate was adjusted to ensure efficient desolvation and transfer of ions into the mass spectrometer, enhancing signal intensity without causing excessive fragmentation. These parameters were fine-tuned through systematic testing, allowing for high throughput and reliable detection of safflower and turmeric powder in saffron samples.

*Full MS:* Full MS scan experiments were singly performed on saffron, safflower and turmeric extract on the EVOQ triple quadrupole. The MS settings were the following: scan mode: Q1MS; polarity: positive; Q1 first mass: 100; Q1 last mass 600; Q1 resolution: 0.7.

*Precursor ion monitoring*: Four precursors for each contaminant were selected based on the full MS analysis and monitored as follows: scan mode: precursor; polarity: positive; Q1 and Q3 resolution: 1.

*Product ion scan* experiments at variable collision energies were carried out with the final aim to optimize the CE value in order to generate the most intense fragmentation pattern from the precursor ions previously selected. The MS settings were the following: scan mode: product; polarity: positive; Q1 and Q3 resolution: 2; collision energy (eV): tested 5–40.

*Multiple reaction monitoring* experiments were performed in order to optimize the final conditions to obtain the most stable and sensitive transitions and relative fragments to be used as quantitative and qualitative markers for the detection of safflower and turmeric in saffron. The MS settings were the following: scan mode: MRM; polarity: positive: for safflower Q1 [446.4], [116.2] and Q3 [116.0], [70.1], respectively; for turmeric Q1 [368.9], [339.0] and Q3: [285.6], [177.7], respectively; for caffeine Q1 [195.0] and Q3 [138.0]; Q1 and Q3 resolution: 2; collision energy (eV): 15

## 3. Results and Discussion

### 3.1. Selection of Safflower and Turmeric Markers and Method Optimization

**Figure 2:**
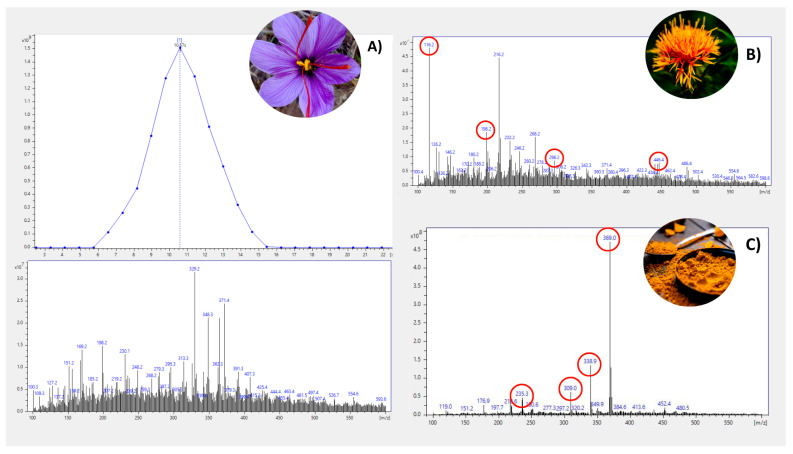
Full scan MS spectra obtained in the mass range 100–600 *m*/*z* in pure saffron (**A**), safflower (**B**) and turmeric (**C**).

In a typical experimental setup, the sample is extracted with a solvent and a small aliquot is deposited on a sample holder placed between the exit of the DART source and the mass spectrometer (MS) inlet. The molecules present in the sample are first thermally desorbed and, due to the interaction with metastable species, generated in the DART source, and positive or negative ions are formed and subsequently analysed by mass spectrometry [34]. By working in the positive ion mode, using helium as the chosen gas, the predominant ions observed by DART-MS are [M+H]^+^ and [M-H]^−^ adducts, respectively, in positive and negative ion mode [35]. Full MS scan experiments in the mass range 100–600 *m*/*z* on safflower and turmeric extracts, prepared according to the method described in Section 2.1, enabled us to generate a list of ions for each extract analysed. The list of ions obtained for each adulterant were compared with those retrieved from the mass spectrum of saffron extract to scout for a shortlist of potential discriminant compounds, to be used ultimately as potential markers for authenticity purposes, as reported in Figure 2. From a list of discriminant molecules, a shorter list was drawn, specifically ions 116.2; 198.2; 296.2; 446.4 for safflower and 369.0; 338.9; 309.0; 235.3 for turmeric.

To investigate the reliability of the pre-selected ions as potential discriminating ions capable of detecting adulteration by safflower and turmeric, saffron extracts fortified with increasing volumes of safflower and turmeric extracts (covering different fortification levels) were prepared and precursor ion-monitoring MS mode was applied to verify the proportional increase of the molecular ion peak with the increase of fortification level.

Ions 116 and 446 were picked out from the pre-identified list of molecular ions, being the most intense and discriminating ions, and the combination precursor ions/fragments 116/70 and 446/116 were selected as the best qualifier and quantifier ions for safflower determination in saffron.

Likewise in the case of turmeric adulteration, transitions 339/178 and 368/286 were revealed to be the most sensitive transitions, and specifically, 339/178 was the precursor/fragment selected as quantitative marker for turmeric detection in saffron.

Experiments based on product ion-monitoring mode were directed to identify the collision energy among the values tested, comprising the values 5, 10, 15, 20, and 40 eV, confirming that 10 eV was the best compromise in terms of fragment abundance and signal stability over repetitive analysis in terms of precursor/fragment ion ratio.

### 3.2. Calibration Curves in Artificially Fortified Saffron Extracts and Repeatability Study

As proof of principle, in order to obtain a calibration curve in the real matrix, safflower was extracted according to the method described above (see Section 2.1 and Section 2.2) and diluted down with saffron extract to cover the concentration range 10-20-30-50-70% (*w*/*w*) of safflower extract mixed with saffron extract by analysing each level in 5 replicates.

In both cases, calibrations were constructed by plotting or not plotting the peak areas of the quantitative transition against the IS peak areas. This enabled the calculation of the equation of the regression curves corrected or not with the IS signal. Comparing each of the equations of the regression curves obtained with and without IS correction resulted in a marked correction of the regression coefficient that varied from R^2^ 0.92 to 0.97 (with and without correction, respectively). Repeatability of the analysis was assessed in saffron extracts fortified with safflower by analysing 10 replicates for each concentration at both 50% and 20% safflower fortification with and without IS correction. CVs obtained were respectively 11% and 17% without correcting with IS and 6 and 8% by peak area correction with IS. Intermediate precision was also assessed by performing five independent replicated analyses of saffron adulterated at 50% during three consecutive days with a calculated CV of 15% with IS correction.

In general, the introduction of caffeine as IS proved to improve the overall variability of the analysis thanks to the compensation effect for the instrumental fluctuation or signal variability due to the sample deposition on the well plate.

Due to the promising results and the better correlation coefficients obtained after normalizing the values of calibration curve for the respective IS peak areas, this approach was adopted for the following investigations about analysis of safflower and turmeric in saffron samples.

### 3.3. Production of Matrix-Matched Calibration Curves in Saffron Powder Recovery Assessment and Analysis of Real Samples

As reported in the Section 2.3 of the Materials and Methods section, saffron samples were adulterated at different concentration levels with safflower or turmeric powder, spanning the concentration range between 5% and 50%. The adulterated powdered samples were extracted as previously described and submitted to DART-MS/MS analysis monitoring markers transitions tracing for turmeric and safflower in saffron along with the IS transition in the developed multiple reaction monitoring (MRM) method previously described.

**Figure 3:**
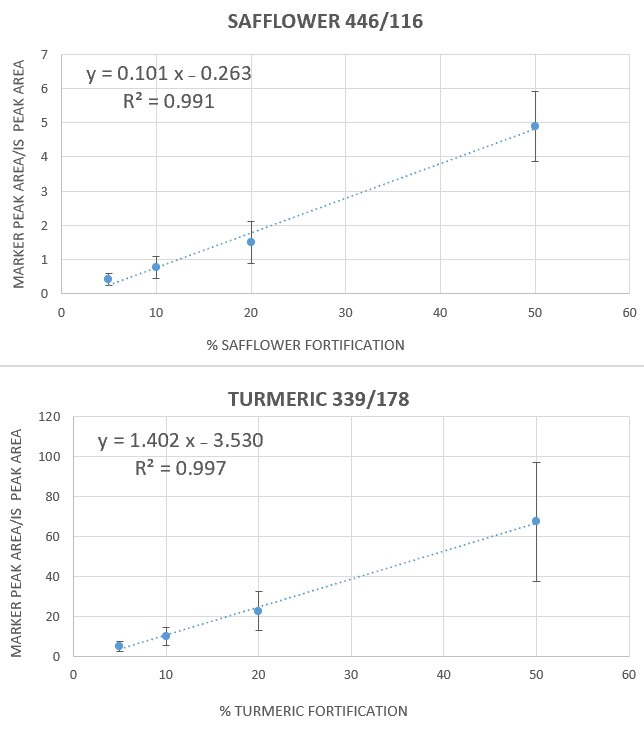
Calibration curves obtained in saffron samples adulterated with either safflower or turmeric powder by monitoring transition 446/116 (for saffron) and 339/178 (for turmeric), corrected by caffeine transition 195/138.

As a result, a calibration curve was constructed for each type of adulterant in saffron. Regression line equations of calibration curves were obtained both for safflower and turmeric adulteration, as shown in Figure 3.

The respective parameters of the regression lines equations along with the LOD and LOQ calculated (3 × sd intercept/slope and 10 × sd intercept/slope) for safflower and turmeric are reported in Table 1.

As shown in Table 1, transition 339/178 proved to be a suitable marker for the detection of tiny amounts of turmeric adulterating saffron, reaching a LOD as low as 3% (*w*/*w*), whereas transition 446/116 proved to be the best candidate transition for the detection of safflower adulterant in saffron, reaching an LOD of 5% (*w*/*w*).

Recovery experiments were carried out by spiking the saffron extract at the level of 20% for each adulterating spice and by comparing final areas with the same level of adulteration realized on the saffron powder before extraction. Recovery experiments were performed on three replicated samples at 20% level each, and recoveries were in the range of 66 ± 5% for marker 446 (tracing for safflower powder) and 53 ± 4% for marker 339 (tracing for turmeric powder).

Finally, five real saffron samples, of which one was authentic and four were adulterated either with unknown amounts of safflower or turmeric, were analysed by the MRM method herein developed. This method was based on the simultaneous monitoring of transitions 339/178, 446/116, and 196/138 for adulterants detection in saffron samples, requiring less than 30 s per sample (MS acquisition time). By interpolating the obtained MS signals of the blind saffron samples acquired with the previously generated calibration curve (for safflower and turmeric), the final percentage of adulteration calculated in the five samples analysed were found to be respectively 3% and 7% for turmeric and 5% and 14% for safflower, while the authentic saffron was found free of any trace of turmeric or safflower. The results obtained confirmed that all adulterated samples were correctly identified by using this method.

### 3.4. Greenness Assessment

The greenness of a method, encompassing aspects such as operator safety and environmental impact of analytical methods, can be assessed by using dedicated tools recently launched to promote working under green analytical chemistry principles [36,37]. Among the different software programs so far developed, the Analytical GREEnness calculator (AGREE) (version v0.5 beta) was used to assess the greenness of the developed method. The input criteria used refer to the 12 significance principles and can be assigned different weights that allow for a certain flexibility. The input criteria refer to material requirements (both quality and quantity), waste generation, energy consumption, the safety of the analyst, and the general approach to the analytical procedure, such as the number of pretreatment steps and location of the analytical device in relation to the object of investigation. Each of the 12 input variables is transformed into a common scale in the 0–1 range, as described in the Principles of Green Analytical Chemistry section by Pena-Pereira et al. [38].

**Figure 4:**
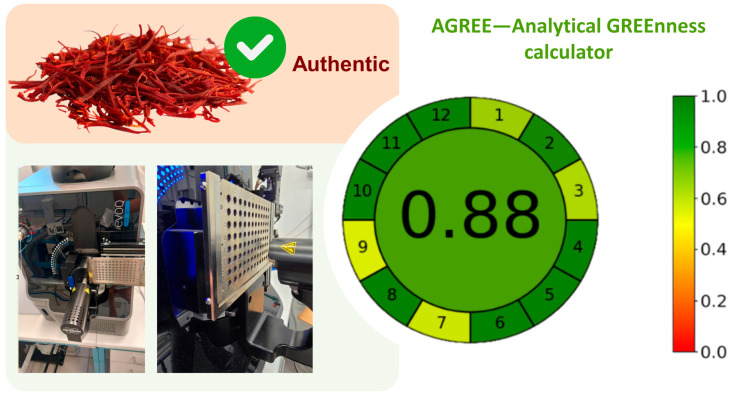
Greenness assessment of the DART-MS/MS method (**left**) and the corresponding colour scale for reference (**right**) by using the AGREE software.

This approach is intended to provide a comprehensive, flexible, and straightforward assessment approach to evaluate the greenness of a method based on significance Green Analytical Chemistry principles to push for methodologies that might be considered green and sustainable. By running the algorithm based on the 12 mentioned criteria of the DART-MS/MS method developed, the final score of 0.88 was obtained, indicating its greenness, as shown in Figure 4. The overall score is typically shown in the middle of the pictogram with values close to 1 (1 is the highest value reachable) and the dark green colour indicating that the assessed procedure is greener. As expected, some steps of the present method might be further improved to increase the overall greenness score, such as minimizing sample prep, performing in situ and high-throughput measurements or reducing volume waste.

### 3.5. Limitations and Future Perspectives

While the developed DART-TQ-MS method demonstrates high sensitivity, selectivity, and speed for the detection of safflower and turmeric adulteration in saffron, several limitations should be acknowledged. First, the method currently targets only two specific adulterants; as a consequence, its applicability to other common adulterants remains to be investigated and demonstrated. Given the diverse chemical nature of potential adulterants, additional efforts in method development—specifically concerning optimization of MRM transitions and ionization parameters—will be required to broaden the method’s scope.

Moreover, the performance of the method across saffron samples of different geographical origins or processing conditions has not yet been systematically assessed. Variability in matrix composition may influence ionization efficiency or introduce matrix effects that could affect quantification accuracy.

Future work will be directed to extend the method, covering a wider range of adulterants and validating its robustness across a broad set of saffron samples collected from the market. In addition, the potential application of the DART-TQ-MS platform to other food or botanical matrices for the detection of contaminants—such as pesticides, illegal dyes, or process-related residues—represents a promising avenue for future investigations. These developments would further enhance potentials of the method as a versatile, high-throughput screening tool for ensuring food safety and authenticity in both regulatory and industrial contexts.

## 4. Conclusions

This paper describes, for the first time, the development of a fast, robust and high-throughput screening method for the quantitative analysis of saffron adulterated with safflower or turmeric powder. The method developed was based on the coupling between the ambient pressure technique DART and the EVOQ triple quadrupole mass spectrometer. Multiple transitions, along with the same acquisition run, were monitored with a total acquisition time of 30 s per analysis. The devised multiple reaction monitoring method included one quantitative and one qualitative transition for each adulterant, along with the transition of caffeine chosen as Internal Standard, to compensate for any instrumental fluctuation. The sample preparation herein optimized was straightforward, user friendly, highly reproducible and proved to significantly reduce the whole analytical pipeline, enabling it to run over 20 samples in 10 min with a significant reduction of organic waste. The method demonstrated to be green according to the AGREE score obtained and was very appealing for the rapidity of analysis execution due to the absence of any chromatographic separation.

Summarizing, the proposed DART-TQ-MS method might represent a practical solution for regulatory authorities and analytical laboratories, particularly applicable for rapid food-fraud screening. Its speed and simplicity might support timely decision-making in import control, market surveillance, and authenticity verification, thereby enhancing the efficiency and responsiveness of routine monitoring activities.

## Figures and Tables

**Table 1:** Regression line parameters, LOD, and LOQ for safflower and turmeric; (x = peak area/% of the adulterant in saffron powder.)

Ion Transition	Calibration Curve Equation	R^2^	LOD (% *w*/*w*)	LOQ (% *w*/*w*)
Safflower in saffron				
116/70	y = 0.0717 (±0.0063) x − 0.1786 (±0.174)	0.985	7	24
**446/116**	y = 0.1014 (±0.0067) x – 0.2633 (±0.185)	0.991	5	18
Turmeric in saffron				
**339/178**	y = 1.4027 (±0.052) x – 3.5298 (±1.445)	0.997	3	10
368/285	y = 4.2545 (±0.253) x − 5.9038 (±0.965)	0.993	5	16

## Data Availability

The original contributions presented in this study are included in the article. Further inquiries can be directed to the corresponding author(s).
